# Comprehensive assessment and meta-analysis of the association between CTNNB1 polymorphisms and cancer risk

**DOI:** 10.1042/BSR20171121

**Published:** 2017-11-23

**Authors:** Yanke Li, Fuqiang Zhang, Dehua Yang

**Affiliations:** Department of Anorectal Surgery, The First Affiliated Hospital of China Medical University, Shenyang, Liaoning 110001, China

**Keywords:** CTNNB1, Cancer, Polymorphism

## Abstract

CTNNB1, encoding β-catenin, is a well-known tumor-related gene in the wnt signaling pathway. It has been reported that CTNNB1 polymorphisms are associated with cancer risk. However, the data were inconsistent. In this article, we conducted a systematic review for the researches related to the association of single nucleotide polymorphisms (SNPs) in CTNNB1 with overall cancer risk. Meanwhile, a series of inclusion and exclusion criteria were set to select articles for quantitative analysis. Consequently, eight case-control studies containing 4388 cases and 4477 controls were included in a meta-analysis of four highly studied CTNNB1 SNPs (rs1798802 A/G, rs4135385 A/G, rs11564475 A/G, and rs2293303 C/T). The association between each SNP and cancer risk was estimated by calculating odds ratios (ORs) and their 95% confidence intervals (95%CIs). The results showed rs1798802 (AA compared with GG: *P*=0.044, OR=0.72) and rs2293303 (TT compared with CC: *P*=0.002, OR=2.86; recessive model: *P*=0.006, OR=2.91; T compared with C: *P*=0.004, OR=1.19) polymorphisms were associated with overall cancer risk. In stratified analysis, rs4135385 polymorphism was found to elevate the risk in Caucasian or in gastrointestinal cancer subgroup. Additionally, rs2293303 conferred to an increased cancer risk when the source of control groups was hospital-based (HB). In conclusion, the three CTNNB1 SNPs were suggested to have the potential to be novel biomarkers for risk prediction of cancer in overall population or some specific subgroups. Our study could provide research clues for further related investigations.

## Introduction

The Wnt signaling pathway was primarily identified for its role in cancer development, which induced the expression of tumor-related genes and contributed to cancer progression by promoting the stabilization of cytoplasmic β-catenin [1,2]. β-catenin, encoded by the *CTNNB1* gene, has two roles in the cells. It forms a functional cadherin–catenin adhesive complex involved in cell–cell adhesion in the membrane, while its nuclear pool participates in signaling pathways and regulates a remarkable variety of cellular process such as cell proliferation, cell survival, and migration [3]. Deregulated β-catenin has been suggested to be related to the development of multiple cancers [4–6].

As the most common form of genetic variation, the single nucleotide polymorphisms (SNPs) are universally present in CTNNB1, which have been extensively investigated. It has been found that mutations in CTNNB1 exons could lead to impaired degradation of β-catenin protein and thus constitutive activation of the Wnt pathway [7,8]. Mounting evidence have also revealed that the somatic mutations in CTNNB1 are often associated with the up-regulation of β-catenin and the pathogenesis of multiple tumors [9,10]. With in-depth basic investigation of CTNNB1 SNPs, accumulating studies have focused on the association between them and the susceptibility to cancer. However, the data were inconsistent. For example, one research reported that rs4135385 was linked to an increased breast cancer (BC) risk [11], but another one suggested this polymorphism could reduce BC risk [12].

In the present study, we systematically reviewed the relationship between CTNNB1 SNPs and overall cancer risk. Based on that, available data were used to perform a meta-analysis, aiming to explore the association of CTNNB1 polymorphisms with cancer and to provide research clues for screening novel biomarkers for cancer risk prediction.

## Materials and methods

### Publication search

A literature search of PubMed and Web of Science was performed by two independent investigators (Yanke Li and Fuqiang Zhang) up to June 12, 2017, with the following keywords: ‘CTNNB1/beta-catenin/β-catenin’; ‘polymorphism/SNP/variant/variation’; and ‘cancer/carcinoma/tumor/neoplasm’. All the studies we selected met these criteria: (i) case–control study; and (ii) based on the association between CTNNB1 SNPs and cancer risk. The exclusion criteria consisted of: (i) duplicate studies; (ii) not related to carcinoma or CTNNB1 SNPs; (iii) no available data and failing to contact with the authors.

### Data extraction

Two investigators (Yanke Li and Fuqiang Zhang) independently extracted data and reached consensus regarding all the items. The following information was obtained from each article: the first author, publication year, country origin, cancer type, genotyping method, source of control groups (hospital- or population - based), sample size of cases and controls, genotype distributions in case and control groups, and adjusted factors. Meanwhile, we classified ethnicity into Asian and Caucasian. And both gastric cancer (GC) and colorectal cancer were categorized as ‘gastrointestinal cancer’ for stratified analysis.

### Assessment of methodology quality

Two reviewers (Yanke Li and Fuqiang Zhang) evaluated the quality of the selected studies by scoring them independently according to recent meta-analyses [13,14]. Six items were assessed: the representativeness of cases, the source of controls, ascertainment of relevant cancers, sample size, quality control of genotyping methods, and Hardy–Weinberg equilibrium (HWE). The scores ranged from 0 to 10 and studies with quality score less than 5 were excluded from subsequent analysis.

### Statistical analysis

The HWE for the genotype frequencies of CTNNB1 polymorphisms in control groups was evaluated using the chi-square test. The association between each SNP and cancer risk was estimated by calculating odds ratios (ORs) and their 95% confidence intervals (95%CIs). Inter-study heterogeneity was examined with a chi-square based Q statistic test (significance at I^2^>50%). We pooled the results using the fix-effect model when inter-study heterogeneity was absent; otherwise, the random-effect model was selected. Begg’s rank correlation and Egger’s linear regression methods were used to assess the publication bias. Sensitivity analysis was conducted to examine whether the overall findings were robust to one or two outlying studies. The analyses mentioned above were all performed using STATA software, version 11.0 (STATA Corp., College Station, TX, U.S.A.). All the tests were two-sided and *P*<0.05 was considered to be statistically significant. Additionally, the dominant and recessive genetic models were defined as heterozygote + homozygote variant compared with homozygote wild and homozygote variant compared with heterozygote + homozygote wild, respectively.

## Results

### Characteristics of the enrolled studies

**Figure 1:**
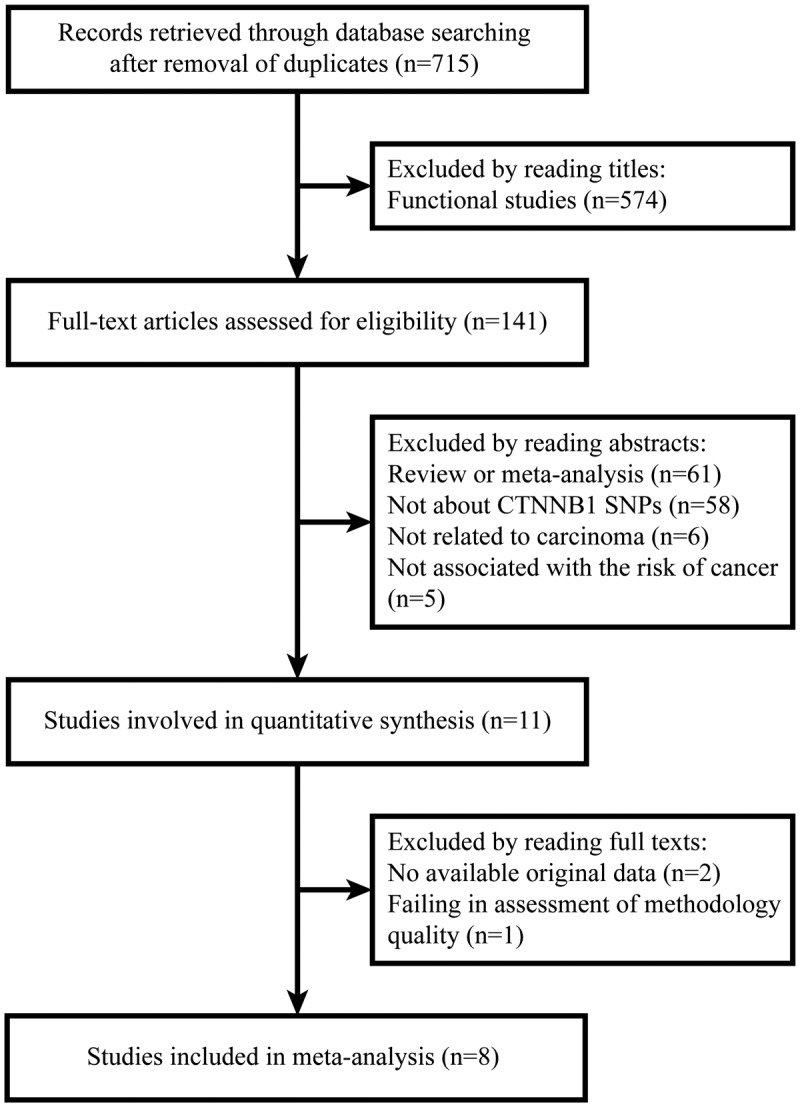
The flow chart of identification for studies included in the meta-analysis

After removal of duplicate studies, a total of 715 records were retrieved through database searching. We excluded 704 records by reading titles and abstracts for the following reasons: 574 were functional studies; 61 were reviews or meta-analyses; 58 were not about CTNNB1 SNPs; 6 were not related to carcinoma; and 5 were not associated with the risk of cancer. Then, 11 case–control studies that met our inclusion criteria were involved in the quantitative synthesis. However, two of them had no available original data and one failed in assessment of methodology quality. Therefore, eight studies containing 4388 cases and 4477 controls were included in our meta-analysis (Figure 1). Their characteristics were shown in Table 1. According to the source of control groups, six studies were hospital-based (HB) and two studies were population-based (PB). The genotype frequency distributions of CTNNB1 SNPs were presented in Table 2. Several records were removed from our meta-analysis due to their genotype frequency distributions in control groups not being in accordance with HWE (*P*_HWE_<0.05). Polymorphisms based on one single study were also excluded. Consequently, four CTNNB1 SNPs were involved in our final calculation, including rs1798802 A/G, rs4135385 A/G, rs11564475 A/G, and rs2293303 C/T.

**Table 1:** The characteristics of enrolled studies

Ref. No.	Year	Country	Ethnicity	Sample size		Source of controls	Genotyping method	Adjusted factors	Quality score	Citation
				Case	Control					
1	2010	China	Asian	307	371	HB	MALDI-TOF	Age	8	[21]
2	2012	China	Asian	944	848	HB	TaqMan	Age and sex	6	[9]
3	2013	Saudi Arabia	Caucasian	99	93	HB	TaqMan	NM	5	[11]
4	2014	Poland	Caucasian	258	282	HB	HRM/PCR-RFLP	NM	6	[22]
5	2015	China	Asian	1160	1336	PB	TaqMan	Age at menarche, age of first birth, and family history of cancer in first-degree relatives	8.5	[10]
6	2016	America	Caucasian	811	814	PB	Illumina’s BeadArray	Age and gender	8.5	[16]
7	2016	South Korea	Asian	245	483	HB	Golder gate	Age and gender	5.5	[23]
8	2016	India	Asian	564	250	HB	PCR-RFLP/ARMS-PCR/ Taqman	Age and gender	6	[24]

Abbreviations: ARMS-PCR, amplification refractory mutation system-PCR; HRM, high-resolution melting curve analysis; NM, not mentioned; PCR-RFLP, PCR-restriction fragment length polymorphism.

**Table 2:** The genotype frequency distributions of CTNNB1 SNPs in studies included

Ref. No.	Year	Cancer type	SNPs^a^	Sample size		Case			Control			*P*_HWE_	Included in meta-analysis
				Case	Control	Homozygote wild	Heterozygote	Homozygote variant	Homozygote wild	Heterozygote	Homozygote variant		
1	2010	Prostate cancer	rs4016435 G/T	307	371	269	37	1	333	37	1	0.979	No^c^
		Prostate cancer	rs1798802 A/G	307	371	27	129	148	33	133	196	0.136	Yes
		Prostate cancer	rs11564459 A/G	307	371	287	20	0	345	26	0	0.484	No^c^
		Prostate cancer	rs11564465 C/T	307	371	184	101	20	232	124	15	0.757	No^c^
		Prostate cancer	rs11564475 A/G	307	371	220	77	5	266	98	7	0.556	Yes
		Prostate cancer	rs2293303 C/T	307	371	222	81	4	277	92	2	0.052	Yes
2	2012	GC	rs1798802 A/G	944	848	106	356	478	72	320	456	0.141	Yes
		GC	rs1880481 A/C	944	848	59	310	573	46	343	459	0.078	No^c^
		GC	rs4135385 A/G	944	848	84	412	448	65	323	460	0.430	Yes
		GC	rs11564475 A/G	944	848	721	197	25	633	204	11	0.228	Yes
		GC	rs2293303 C/T	944	848	728	135	71	647	187	14	0.908	Yes
3	2013	BC	rs13072632 C/T	99	93	9	46	44	10	42	41	0.876	No^c^
		BC	rs4135385 A/G	99	93	63	31	5	72	18	3	0.180	Yes
4	2014	Ovarian cancer	rs4533622 A/C	258	282	78	113	37	90	122	70	**0.029**	No^b^
		Ovarian cancer	rs2953 T/G	258	282	37	113	78	70	122	90	**0.029**	No^b^
5	2015	BC	rs4533622 A/C	1160	1336	69	366	725	75	444	817	0.156	No^c^
		BC	rs4135385 A/G	1160	1336	264	601	295	303	677	356	0.582	Yes
		BC	rs2293303 C/T	1160	1336	879	251	30	1048	269	19	0.714	Yes
6	2016	Colorectal cancer	rs4135385 A/G	811	814	460	298	52	503	263	45	0.174	Yes
7	2016	HCC	rs3864004 A/G	245	483	16	69	156	24	165	290	0.932	No^c^
		HCC	rs4135385 A/G	245	483	62	117	64	103	237	143	0.794	Yes
		HCC	ht1_GG +/−	245	483	63	114	59	140	235	103	0.813	No^c^
		HCC	ht2_GA +/−	245	483	21	136	79	27	287	164	**<0.001**	No^b^
8	2016	Gall bladder cancer	rs4135385 A/G	564	250	327	179	58	155	76	19	**0.031**	No^b^

^a^, the ancestral alleles were referenced in the NCBI database; ^b^, excluded due to the SNP not being in accordance with HWE; ^c^, excluded due to the limited number for this locus. The results are in bold if *P*<0.05. Abbreviations: HCC, hepatocellular carcinoma; *P*_HWE_, the *P* value for HWE in control groups.

### Quantitative data synthesis of four CTNNB1 SNPs

First, we calculated the pooled ORs of all enrolled studies to estimate the association between the four SNPs in CTNNB1 and overall cancer risk. The rs1798802 and rs2293303 polymorphisms were found to be associated with cancer risk, while the rs4135385 and rs11564475 polymorphisms did not demonstrate remarkable association in overall population. For rs1798802, the variant type GG significantly decreased the risk when compared with the wild type AA (*P*=0.044, OR=0.72, 95%CI=0.52–0.99). For rs2293303, its variant genotype, recessive and allelic models were all associated with an increased cancer risk (TT compared with CC: *P*=0.002, OR=2.86, 95%CI=1.45–5.61; recessive model: *P*=0.006, OR=2.91, 95%CI=1.35–6.26; T compared with C: *P*=0.004, OR=1.19, 95%CI=1.06–1.34, Table 3).

**Table 3:** Meta-analysis of the association between CTNNB1 polymorphisms and cancer risk

SNPs	N	Heterozygote compared with homozygote wild			Homozygote variant compared with homozygote wild			Dominant model			Recessive model			Allelic model		
		*P*	OR (95%CI)	I^2^ (%)	*P*	OR (95%CI)	I^2^ (%)	*P*	OR (95%CI)	I^2^ (%)	*P*	OR (95%CI)	I^2^ (%)	*P*	OR (95%CI)	I^2^ (%)
**rs1798802 A/G**	2	0.790^a^	0.94 (0.58–1.51)	63.0	**0.044**	0.72 (0.52–0.99)	0.0	0.753^a^	0.92 (0.55–1.54)	69.5	0.221	0.89 (0.74–1.07)	0.0	0.125	0.90 (0.78–1.03)	48.2
**rs4135385 A/G**	5	0.969^a^	0.99 (0.74–1.34)	81.3	0.232^a^	1.40 (0.81–2.45)	86.0	0.652^a^	1.05 (0.86–1.27)	62.6	0.204^a^	1.40 (0.83–2.34)	87.0	0.310^a^	1.08 (0.93–1.26)	67.5
Ethnicity																
Asian	3	0.205^a^	0.82 (0.60–1.12)	75.9	0.418^a^	1.42 (0.61–3.34)	92.7	0.321	0.94 (0.82–1.07)	0.0	0.302^a^	1.49 (0.70–3.20)	93.4	0.962^a^	1.00 (0.85–1.18)	63.4
Caucasian	2	**0.012**	1.29 (1.06–1.58)	39.8	0.195	1.30 (0.87–1.95)	0.0	**0.007**	1.30 (1.07–1.57)	44.3	0.376	1.20 (0.81–1.78)	0.0	**0.011**	1.23 (1.05–1.43)	45.4
Cancer type																
Gastrointestinal cancer	2	0.735^a^	0.90 (0.47–1.71)	93.8	0.184^a^	2.35 (0.67–8.26)	91.9	0.671^a^	1.07 (0.79–1.45)	76.1	0.237^a^	2.36 (0.57–9.75)	93.7	**0.006**	1.19 (1.05–1.35)	0.0
BC	2	0.404^a^	1.31 (0.70–2.44)	70.5	0.769	0.97 (0.77–1.21)	0.0	0.427^a^	1.30 (0.68–2.49)	75.1	0.545	0.95 (0.79–1.13)	0.0	0.475^a^	1.23 (0.70–2.17)	76.7
HCC	1	0.313	0.82 (0.56–1.21)	NA	0.178	0.74 (0.48–1.15)	NA	0.204	0.79 (0.55–1.14)	NA	0.357	0.85 (0.60–1.20)	NA	0.179	0.86 (0.69–1.07)	NA
Source of controls																
HB	3	0.768^a^	0.93 (0.56–1.55)	79.3	0.392^a^	1.83 (0.46–7.33)	91.8	0.926^a^	1.02 (0.70–1.49)	67.1	0.359^a^	1.89 (0.49–7.31)	92.4	0.434^a^	1.14 (0.82–1.58)	76.0
PB	2	0.128	1.12 (0.97–1.29)	43.7	0.889	1.01 (0.83–1.24)	27.0	0.349^a^	1.11 (0.89–1.38)	60.5	0.736	0.97 (0.83–1.15)	0.0	0.519^a^	1.07 (0.88–1.29)	73.8
**rs11564475 A/G**	2	0.169	0.88 (0.73–1.06)	0.0	0.124	1.60 (0.88–2.90)	30.9	0.353	0.92 (0.76–1.10)	0.0	0.099	1.65 (0.91–2.99)	35.1	0.718	0.97 (0.82–1.14)	0.0
**rs2293303 C/T**	3	0.657^a^	0.92 (0.63–1.34)	84.6	**0.002**^a^	2.86 (1.45–5.61)	54.7	0.375	1.06 (0.93–1.21)	30.5	**0.006**^a^	2.91 (1.35–6.26)	64.3	**0.004**	1.19 (1.06–1.34)	0.0
Source of controls																
HB	2	0.480^a^	0.83 (0.49–1.40)	83.7	**<0.001**	4.28 (2.47–7.42)	0.0	0.749	0.97 (0.81–1.17)	5.6	**<0.001**	4.61 (2.67–7.98)	0.0	**0.043**	1.18 (1.01–1.39)	0.0
PB	1	0.281	1.11 (0.92–1.35)	NA	0.033	1.88 (1.05–3.37)	NA	0.113	1.16 (0.97–1.40)	NA	0.039	1.84 (1.03–3.29)	NA	0.041	1.19 (1.01–1.41)	NA

^a^, *P* was calculated by random model. The results are in bold if *P*<0.05.

Due to the existence of heterogeneity, stratified analysis was performed on the basis of ethnicity, cancer type, and source of controls. The rs4135385 and rs2293303 polymorphisms were found to be associated with cancer susceptibility in some specific subgroups. In Caucasian population, rs4135385 could elevate the risk of overall cancer in heterozygote genotype, dominant, and allelic models (AG compared with AA: *P*=0.012, OR=1.29, 95%CI=1.06–1.58; dominant model: *P*=0.007, OR=1.30, 95%CI=1.07–1.57; G compared with A: *P*=0.011, OR=1.23, 95%CI=1.05–1.43). Its variant G allele was also observed to increase the gastrointestinal cancer risk when compared with the wild A allele (*P*=0.006, OR=1.19, 95%CI=1.05–1.35). For rs2293303, the variant type TT, recessive and allelic models conferred an elevated risk when the control groups were HB (TT compared with CC: *P*<0.001, OR=4.28, 95%CI=2.47–7.42; recessive model: *P*<0.001, OR=4.61, 95%CI=2.67–7.98; T compared with C: *P*=0.043, OR=1.18, 95%CI=1.01–1.39, Table 3).

### Sensitivity analysis

We subsequently conducted sensitivity analysis to explore the influence of one individual study on the pooled results by estimating the ORs and 95%CIs before and after removal of one record from meta-analysis. No outcome was found to range from insignificant to statistically significant after any individual study was removed (Supplementary Table S1).

### Publication bias

Begg’s test and Egger’s test were used to evaluate the potential publication bias of the included studies. No significant publication bias was indicated in any genetic model of the studied CTNNB1 SNPs (Table 4).

**Table 4:** The results of Begg’s and Egger’s test for the publication bias

Comparison type	Begg’s test		Egger’s test	
	Z value	*P* value	t value	*P* value
**rs1798802 A/G**				
Heterozygote compared with homozygote wild	1.00	0.317	NA	NA
Homozygote variant compared with homozygote wild	1.00	0.317	NA	NA
Dominant model	1.00	0.317	NA	NA
Recessive model	1.00	0.317	NA	NA
Allelic model	1.00	0.317	NA	NA
**rs4135385 A/G**				
Heterozygote compared with homozygote wild	0.00	1.000	0.23	0.833
Homozygote variant compared with homozygote wild	1.47	0.142	1.07	0.364
Dominant model	–0.49	0.624	0.40	0.715
Recessive model	1.96	0.050	1.24	0.303
Allelic model	0.49	0.624	1.09	0.356
**rs11564475 A/G**				
Heterozygote compared with homozygote wild	1.00	0.317	NA	NA
Homozygote variant compared with homozygote wild	–1.00	0.317	NA	NA
Dominant model	1.00	0.317	NA	NA
Recessive model	–1.00	0.317	NA	NA
Allelic model	–1.00	0.317	NA	NA
**rs2293303 C/T**				
Heterozygote compared with homozygote wild	–0.52	0.602	–0.15	0.906
Homozygote variant compared with homozygote wild	0.52	0.602	–0.08	0.949
Dominant model	–0.52	0.602	–0.08	0.952
Recessive model	0.52	0.602	–0.10	0.938
Allelic model	–0.52	0.602	–2.48	0.244

The results are in bold if *P*<0.05. Abbreviation: NA, not available.

## Discussion

Aberrant activation of the Wnt signaling pathway has been found in many tumors, caused by the accumulation of CTNNB1 expression product in cell [15]. It is well accepted that genetic variations influencing the expression and/or function of CTNNB1 might be related to the susceptibility to cancer. In the present study, we collected relevant, published articles and available data. A meta-analysis was performed for the association between four highly studied CTNNB1 SNPs and cancer risk, including rs1798802 A/G, rs4135385 A/G, rs11564475 A/G, and rs2293303 C/T. The results showed that three SNPs were associated with cancer risk other than rs11564475. To the best of our knowledge, it is the first systematic review in this field, and also the first time that the four CTNNB1 SNPs are reported in a meta-analysis.

Regarding rs1798802, our findings differed from other related studies to some extent. We observed that the variant type of rs1798802 could reduce the risk of overall cancer, suggesting a potential predictive ability of this polymorphism for cancer risk. The SNP is located in the intron region of CTNNB1, thus it might affect gene transcription and shearing, alter CTNNB1 expression level, and exert effects on the downstream caner-related molecules [14]. The original data of rs1798802 were extracted from two case–control studies. However, no association between the SNP and cancer risk was found in any of them. From our perspective, this phenomenon might result from the limited sample size, ethic diversity of populations, and complicated environmental factors. Therefore, further studies concentrated on this SNP are needed to be involved in meta-analysis for timely updating and obtaining more reliable results.

Currently, rs4135385 is the most intensively investigated polymorphism among all the CTNNB1 SNPs, recording the largest number of reports in this field. Several researches have demonstrated that rs4135385 is associated with multiple cancers [9,16]. As the polymorphism is located in the intron 13 of CTNNB1, it is conceivable to affect RNA splicing and thus aberrant β-catenin expression [17]. Another possible mechanism is that the functional variant responsible for the observations is not the analyzed SNP rs4135385 but another unknown variant in linkage disequilibrium (LD) with it, as illustrated in previous studies [18]. Very interestingly, our study showed the SNP could only elevate the risk in the subgroups of Caucasian and gastrointestinal cancer rather than overall population. To explain the phenomenon that occurred in different subgroups, we searched the NCBI dbSNP database (https://www.ncbi.nlm.nih.gov/projects/SNP/snp_ref.cgi?rs=4135385) to obtain the genotype frequency distributions of rs4135385 among different ethnic populations: A/A 48.3%, A/G 46.7%, and G/G 5.0% for European; A/A 17.8%, A/G 57.8%, and G/G 24.4% for Asian. Obviously, the risk genotypes frequency of the SNP is higher in Asian than in Caucasian. Therefore, it is reasonable that the effects of the polymorphism on cancer susceptibility might be masked by the higher frequency of risk genotypes in healthy subjects. Additionally, it has been reported that the association of rs4135385 with GC risk is more prominent among patients with cardia GC than non-cardia GC [9]. Similar site-specific differences could also be observed in our results, which may be partially attributed to the biological discrepancy between different cancers [19], although the exact mechanism remains to be elucidated. Therefore, rs4135385 could be used to determine cancer risk in Caucasian or to predict gastrointestinal cancer risk.

Rs2293303 was the only one found to be associated with cancer risk both in overall and stratified analysis among the studied SNPs. Importantly, it is a synonymous SNP (sSNP), located in gene-coding regions of CTNNB1 [10]. Although sSNPs do not change the amino acid composition of the encoded proteins owing to the degeneracy of the genetic code, considerable evidence has accumulated to demonstrate that synonymous substitutions could affect mRNA splicing, mRNA stability, splicing accuracy, mRNA structure, translation fidelity, thus protein expression and enzymatic activity [20]. In addition, sSNPs can also influence protein folding and conformation because tertiary protein structure could be affected by codon bias [20], therefore, they have functional and clinical consequences. Moreover, the association of the SNP with cancer was more remarkable in the HB subgroup. The HB controls mainly consist of individuals who initiatively seek for physical examination in hospitals, thus they are likely to have higher educational levels than PB subjects, which may account for that phenomenon. In conclusion, rs2293303 could also be a predictive biomarker for cancer risk.

Several limitations in our study should be acknowledged. First of all, only English documents were searched while reports in other languages were not involved, which may lead to publication bias. In addition, the study about the association of CTNNB1 polymorphisms with cancer risk remains a relatively emerging field; consequently, the relevant researches are lacking. Besides, the records for which *P*_HWE_<0.05 were all excluded from final calculation. These conditions may have led to the limited number of records included in our meta-analysis.

In summary, we systematically reviewed the relationship between CTNNB1 polymorphisms and overall cancer risk. Meanwhile, available data was used to perform a meta-analysis for four highly studied SNPs. The results suggested three of them were associated with cancer risk in overall population or some specific subgroups, including rs1798802, rs4135385, and rs2293303. Our study could provide research clues for further investigations focused on the identification of novel biomarkers with cancer forewarning function.

## Supporting information

**Table S1:** ORs (95%CIs) of sensitivity analysis

## Abbreviations

BC: breast cancer
GC: gastric cancer
HB: hospital-based
OR: odds ratio
PB: population-based
SNP: single nucleotide polymorphism
sSNP: synonymous single nucleotide polymorphism
CI: confidence interval
