# Supplementary material for: Comprehensive assessment and meta-analysis of the association between CTNNB1 polymorphisms and cancer risk
Source: Biosci Rep. 2017 Nov 23;37(6):BSR20171121. doi: 10.1042/BSR20171121 (PMC5700267; doi:10.1042/BSR20171121)
Supplement: Supplementary file 1 [file bsr20171121_Supp1.pdf]

Table S1. ORs (95%CIs) of sensitivity analysis

Excluding literature one by one	Heterozygote vs. Homozygote wild OR (95%CI)	Homozygote variant vs. Homozygote wild OR (95%CI)	Dominant model OR (95%CI)	Recessive model OR (95%CI)	Allelic model OR (95%CI)
**rs1798802 A/G**					
Overall	0.94(0.58-1.51)	0.72(0.52-0.99)	0.92(0.55-1.54)	0.89(0.74-1.07)	0.90(0.78-1.03)
Shu-Pin Huang (2010)	0.76(0.54-1.06)	0.71(0.51-0.99)	0.73(0.53-1.00)	0.89(0.74-1.07)	0.87(0.75-1.01)
Shizhi Wang (2012)	1.24(0.76-2.01)	1.24(0.08-19.88)	1.24(0.77-2.00)	1.21(0.08-19.41)	1.22(0.77-1.93)
**rs4135385 A/G**					
Overall	0.99(0.74-1.34)	1.40(0.81-2.45)	1.05(0.86-1.27)	1.40(0.83-2.34)	1.08(0.93-1.26)
Shizhi Wang (2012)	1.11(0.88-1.40)	0.98(0.78-1.23)	1.10(0.86-1.41)	0.95(0.82-1.11)	1.06(0.88-1.27)
Mohammad Saud Alanazi (2013)	0.91(0.68-1.22)	1.36(0.75-2.47)	1.00(0.84-1.19)	1.38(0.80-2.40)	1.05(0.91-1.21)
Yu-Mian Jia (2015)	1.01(0.66-1.54)	1.63(0.71-3.74)	1.08(0.81-1.43)	1.64(0.73-3.66)	1.13(0.93-1.39)
Michelle A.T. Hildebrandt (2016)	0.93(0.66-1.32)	1.48(0.69-3.18)	0.98(0.79-1.21)	1.50(0.75-3.00)	1.06(0.87-1.28)
Soon Sun Kim (2016)	1.04(0.74-1.48)	1.71(0.84-3.47)	1.10(0.89-1.36)	1.66(0.80-3.45)	1.14(0.97-1.35)
**rs11564475 A/G**					
Overall	0.88(0.73-1.06)	1.60(0.88-2.90)	0.92(0.76-1.10)	1.65(0.91-2.99)	0.97(0.82-1.14)
Shu-Pin Huang (2010)	0.85(0.68-1.06)	2.00(0.97-4.09)	0.91(0.73-1.12)	2.07(1.01-4.24)	0.98(0.81-1.19)
Shizhi Wang (2012)	0.95(0.67-1.35)	0.86(0.27-2.76)	0.94(0.67-1.33)	0.88(0.28-2.79)	0.95(0.70-1.28)
**rs2293303 C/T**					
Overall	0.92(0.63-1.34)	2.86(1.45-5.61)	1.06(0.93-1.21)	2.91(1.35-6.26)	1.19(1.06-1.34)
Shu-Pin Huang (2010)	0.85(0.50-1.46)	2.91(1.23-6.90)	1.05(0.91-1.21)	3.00(1.14-7.90)	1.20(1.05-1.36)
Shizhi Wang (2012)	1.11(0.94-1.31)	1.94(1.12-3.36)	1.16(0.98-1.36)	1.89(1.09-3.28)	1.18(1.02-1.37)
Yu-Mian Jia (2015)	0.83(0.49-1.40)	4.24(2.44-7.36)	0.97(0.81-1.17)	4.56(2.63-7.90)	1.18(1.00-1.39)

Note: OR, odds ratio; CI, confidence interval.
